# Access to antiretroviral treatment, issues of well-being and public health governance in Chad: what justifies the limited success of the universal access policy?

**DOI:** 10.1186/1747-5341-8-8

**Published:** 2013-08-01

**Authors:** Jacquineau Azétsop, Blondin A Diop

**Affiliations:** 1 Département de Santé Publique, Faculté des Sciences de la Santé de l’Université de N’djaména, Avenue Mobutu, BP. 1117 N’djaména, Tchad; 2 Hôpital Générale de Référence de N’djaména, Avenue Mobutu, BP. 1117 N’djaména, Tchad

## Abstract

Universal access to antiretroviral treatment (ART) in Chad was officially declared in December 2006. This presidential initiative was and is still funded 100% by the country’s budget and external donors’ financial support. Many factors have triggered the spread of AIDS. Some of these factors include the existence of norms and beliefs that create or increase exposure, the low-level education that precludes access to health information, social unrest, and population migration to areas of high economic opportunities and gender-based discrimination. Social forces that influence the distribution of dimensions of well-being and shape risks for infection also determine the persistence of access barriers to ART. The universal access policy is quite revolutionary but should be informed by the systemic barriers to access so as to promote equity. It is not enough to distribute ARVs and provide health services when health systems are poorly organized and managed. Comprehensive access to ART raises many organizational, ethical and policy problems that need to be solved to achieve equity in access. This paper argues that the persistence of access barriers is due to weak health systems and a poor public health leadership. AIDS has challenged health systems in a manner that is essentially different from other health problems.

## Introduction

Universal access to antiretroviral treatment (ART) in Chad was officially declared in December 2006. This presidential initiative was and is still funded 100% by the country’s budget and external donors’ financial support. Through this major initiative, the government provides free access to first and even second line antiretroviral drugs (ARVs) as well as treatment for opportunistic diseases to people living with HIV (PLWH). The universal access package also includes measures and programs to prevent HIV infection in the general population. In spite of this bold move towards ensuring significant reduction in HIV incidence, access to ART is unevenly distributed. Children, women, people living in hard-to-reach areas, and stigmatized PLWH do not always have sufficient access to ART. Persistence of access barriers challenges justice-minded individuals to question the way social structures distribute dimensions of well-being. Based on Madison Powers’ and Ruth Faden’s understanding of justice as well-being [1], this paper argues that the limited success of the universal access policy in Chad results from the insufficient inclusion of issues related to people’s overall well-being.

Most people who do not have access to ART lack important dimensions of well-being which are often distributed by social institutions. These dimensions of well-being include respect for their human worth, freedom from want, health, ability to avoid preventable death, access to life-saving information, and participation. When segregated by region, age and sex, HIV/AIDS epidemiological data show that the lack of dimensions of well-being determines the likelihood to have access to treatment. The success of the universal access policy is impaired by social inequities coupled with factors such as fragmented health systems and poor public health leadership. Within such a poorly organized a social system, ART access is often constrained by structural forces. Framing the universal access policy within the contexts of both development policy and the need for reshaping health systems will ensure that the social roots of ART inaccessibility and of health systems’ shortcomings can be identified and addressed. The fight against HIV and AIDS cannot be separated from other spheres of social life [2].

## Method

This paper is a systematic review of HIV and AIDS epidemiology data collected in Chad by UNAIDS in 2009, the National AIDS Council in 2010 [3,4] and the Ministry of Public Health (MPH) in 2007 [5]. The evidentiary bases of the claims made in the paper are borrowed from all the aforementioned studies and from the 2009 Draft of Annual Health Statistics produced by the MPH [6]. The studies reviewed in this paper are pioneering research done with limited bibliographic resources and empirical data. In spite of these limitations, the quality of the epidemiological claims contained in these studies gives the accurate picture of the HIV pandemic in Chad.

Unlike Sen’s capability approach, which tends to be appealing but remains too broad to be concrete [7], the ethical framework through which epidemiological data is analyzed presents health justice from the Aristotelian perspective as well-being. This framework is borrowed from the book of Madison Powers’ and Ruth Faden, *Social Justice: The Moral Foundations of Public Health and Health Policy*[1]. Powers and Faden believe that, in a just society, there are essential dimensions of well-being that people should normally have in order to live a decent life and to avoid unnecessary suffering [1]. We use their approach to examine and to understand barriers to ART as raising issues of justice.

### Epidemiological data

The 2005 sero-prevalence survey showed that HIV prevalence was estimated at 3.3% in the entire country. The prevalence was higher in urban (7%) compared to rural (2.3%) populations. The distribution of this data by age was 1.9% in the under 25 age group, 4.6% in the 25–29 age group and 3% in those aged 45–49 [3]. In the male population those in the 25–29 age groups are most affected (3.5%). In the female group, those in the 25 to 29 and 30 to 34 age groups are the most affected (5.6%) [5]. The prevalence was higher among women (4.0%) than among men (2.65%) [5]. HIV infection affects disproportionately young urban women aged 25 to 34 more than any other group. Young adults (25–34) represented the age group with the highest vulnerability [5]. Incident cases within this population are 2 to 3% higher than in any other group. Targeting this group for prevention and treatment preferences is a good strategy for reducing HIV incidence and prevalence in the overall population.

Many factors have triggered the spread of AIDS. Some of these factors include the existence of customary norms and beliefs that create or increase exposure especially among women, the low-level education that precludes access to health information, social unrest, and population migration to areas of high economic opportunities [4].

#### ***Most-at-risk-population groups***

The most-at-risk-population groups (MARPS) include sex workers, women, young people and refugees. The 2010 UNAIDS survey showed that HIV prevalence in vulnerable groups was higher than in the ordinary population. The prevalence in Bol, the main city of the Lake region, in pregnant women increased from 5% in 2002 to 9.5% in 2009, while prevalence among pregnant women in Abéché significantly decreased from 3.9 to 2.5% during the same period [3]. Sex workers constitute the most vulnerable group that requires special attention. The proportion of urban HIV-infected sex workers is quite high: Kélo (27.5%), Léré (22.6%) and N’Djaména (25.5%) [4]. The distribution of HIV according to age within this vulnerable group is estimated at 13.7% in the 30–34 age group and 22% in the 20–24 age group [4].

The proportion of HIV-infected women is high compared to that of men. Women represent 65% of PLWH. The risk of mother-to-child transmission (MTCT) remains high due to limited access to antenatal care. The likelihood is even greater when we know that the integration of prevention of mother-to-child transmission (PMTCT) to antenatal care is still in its infancy [3].

The results of the same survey showed that important socioeconomic and cultural dynamics determine risk differentials between population groups. In the cosmopolitan city of N’Djamena, HIV prevalence is estimated at 8.3%. In the Logone Occidental/Logone Oriental/Tandjilé axis, where there are intense economic activities due the flux of money coming from oil drilling, HIV prevalence (6.4%) is two times higher than the national average. In the Lake region, where there is an intense fishing activity, HIV prevalence is estimated at 8.3% in the general population. These regions are meeting points of people coming from all over the country and from neighboring countries. People from other regions of the country and citizens from neighboring countries migrate to regions of high economic activities or places of intensive fishing, leaving their families behind. Migration of sexually active adults in search of labor is known to be a factor that increases the risk of HIV infection. The spread of the virus also follows the path of civil wars, the circuits of labor migrants, and the flight of refugees.

#### ***Universal access to ART and barriers to access***

Availability of ARVs has significantly improved the prognosis of PLWH through procedures that help control viral replication, reduce viral load, restore immune system function, and extend life. Besides having a positive impact on the quality of life, the treatment is also a possible preventive measure because it reduces viral load which, in turn, reduces the risk of HIV transmission. However, this does not mean that increased treatment coverage should be considered as a substitute for other preventive measures that have been proven to work in reducing new HIV infections. Thus, increased efforts to boost treatment coverage should divert attention from other areas of HIV and AIDS work. Possible efforts to roll out large-scale treatment interventions that would immediately treat HIV-positive individuals is unlikely to do so due to drug resistance, side effects of drugs, and need for patient consent.

Many African countries have been providing free first-line ART to PLWH since 2004. Since 2007, ARVs are freely given to eligible PLWH in Chad. The universal availability of ART has improved treatment access. The number of patients on ARVs increased from 7,215 in 2007 to 32, 832 in 2011 against 97,196 PLWH who actually need to be on ARVs [5]. The proportion of PLWH on ARVs represents 33% coverage of people eligible for treatment. The MARPs are identified as a priority target for the HIV/AIDS response.

The disease itself carries a stigma which affects infected individuals’ social status. In order to avoid being discriminated against, PLWH refrain from seeking treatment or hide their status from their relatives. Stigma and discrimination influence access to ART. Socio-cultural and geographic factors, as well as the weaknesses of health systems, also represent important barriers to access to preventive and curative health services, especially in remote regions. The epidemic is central to the topic of inequality. Not only is HIV-risk perception quite low, but also information that provides individuals with the knowledge of how to access health services is still not available to many people [3]. The chance to survive with an HIV infection is not spread evenly. It is determined by cultural, political, economic, geographic and political factors.

### Justice as well-being

Access to ART raises many organizational, ethical and policy problems. Social forces that influence the distribution of dimensions of well-being [1] also determine the persistence of access barriers. ART accessibility raises issues of social justice with regard to the challenge of ensuring that the treatment or preventive intervention reaches everyone in need. Equitable access is achieved when every individual has equal probability of receiving ART regardless of their social affiliation. Knowing the negative consequences of AIDS, nothing less can satisfy genuine human sensitivity and the human conscience.

Madison Powers and Ruth Faden have developed a non-ideal theory, because inequality of any sort provides a real world context to address issues of justice. Their approach to justice goes beyond mere access to basic goods to include the overall well-being of people in social communities and groups. Powers’ and Faden’s approach is concerned with human flourishing, which can be understood as including many dimensions, each of which represents something of independent moral significance [1]. Distributive principles cannot ensure access to ART in isolation from the social context of HIV infection. Access barriers denote the lack of one or many dimensions of well-being. Far from focusing solely on individuals, this approach to justice places MARPs and PLWH, and how they are doing, at the heart of health policy. The realm of social policy is an important means within which broad solutions to access can be found. Hence, the question of justice does not come to us from examining an individual’s behavior but by questioning social institutions’ ability to create conditions conducive to well-being [1].

Justice requires that society provide every citizen, in one way or another, with the possibility of having a sufficient amount of each of the essential dimensions of well-being. Our commitment to justice attaches a special moral urgency to remediating the conditions of those who are more vulnerable in our society. Each dimension of well-being provides prisms through which the justice of political structures, social practices and individual behavior can be understood and assessed. Our approach to “justice captures what we believe are the twin moral impulses that animate public health: to improve human well-being by improving health and related dimensions of well-being (positive aim of public health) and to do so in particular by focusing on the needs of those who are the most disadvantaged [1].” We, then, reject any attempt to separate spheres of justice as if we can talk about the universal access policy in Chad without talking about the socioeconomic conditions and the socio-cultural practices that make access possible or not [3].

The lack of some dimensions of well-being prevents many people from having what is needed to avoid infection and death. Those who were already marginalized by social forces find it hard to have proper access to ART. Powers and Faden astutely assert that “the disadvantages associated with inequalities in the social basis of well-being in all of its dimensions are various, they often travel in tandem, and they can mutually reinforce and perpetuate one another. In turn, they can and often do affect a whole range of dimensions of well-being [1].” The most important dimensions of justice which are instrumental for accessing ART include the respect for human worth, freedom from want, health, ability to avoid preventable death, and having access to life-saving information.

#### ***Respect for human worth***

Universal access can be “misleading as it is not meant to ‘imply that there will be, or should be, 100 percent utilization by all individuals of every prevention, treatment, care and support intervention.’ Instead, it refers to nationally driven and comprehensive responses in these areas, signifying a concrete commitment and renewed resolve the world over, but particularly within each country, to reverse the course of the epidemic [8].” Concretely, universal access is achieved when more than 80 percent of those in need of ART actually have access to it. Although this target has not been met, with current global treatment coverage at 54 percent, the goal of universal access to HIV treatment remains an important one for Chad, with a new target of universal access by 2015 agreed in 2010 [9]. In the case of infection, is there someone who will be willing to be left aside without treatment? Instead of arguing for a narrow approach to universal coverage, a genuine human mind will argue for a progressive realization of universal coverage.

Access to ART is related to the question of respect, since inability to access the needed care is an important dimension of well-being which, when lacking, interferes with other dimensions of well-being. The underprivileged use public health services the most, not because these services provide them with quality care, but because they cannot afford care in private clinics. Society’s obligation to ensure universal access to healthcare (including ART) rests not only on the effects of access on health but also on what justice requires with regard to what is necessary for a person’s being respected as being endowed with a dignity that cannot be violated [1].

Respect for human worth means treating others as dignified moral beings deserving of equal moral concern. Respect for others requires the ability to see others as independent sources of moral worth [1]. This dimension of well-being finds its core foundation in the dignity which is inherently inscribed in every single human being by the very fact of being human. The intrinsic worth inherent in every human being is grounded in what a human being is as a human being. It is not an attributed or imputed reality but an ontological quality that nothing, no one and no contingent determination can reify. Human dignity or worth, therefore, transcends any social order as the basis for rights and is neither granted by society nor can be legitimately violated by society. All persons carry a dignity that is not diminished by suffering or sickness. In this way, human dignity is the conceptual basis for human rights to treatment and prevention from harm.

The concept of “human dignity” provides the foundation for many normative claims on issues concerning the human person and society. One direct normative implication of human dignity or worth is that every human being ought to be acknowledged as an inherently valuable member of the human community, with an integrated bodily and spiritual nature. This conception of human dignity has normative implications for the understanding of distributive justice, the common good, and the human rights to life and health. Respect for human dignity does not simply refer to the need not to hurt the other person but, more genuinely, it calls for the creation of conditions conducive to human flourishing. Genuine respect for human dignity requires that society alters the root causes of misery and preventable deaths. Our sense of humanity is deeply challenged when less than 50% of eligible PLWH are receiving treatment and the needs of most MARPS are not systematically addressed. The claim of justice challenges us to address all that does not favor the full expression of people’s humanity. Violations of human dignity are perceptible through stigmatization and discrimination of PLWH, access differentials between urban and rural dwellers, and insufficient access of women and children to ARVs.

#### ***Human worth, stigma and discrimination against people living with HIV***

Stigmatizing behavior and discriminatory attitudes undermine the worth of PLWH simply because they are infected with HIV. The National AIDS Council (NAC) survey findings revealed high levels of HIV/AIDS-related stigma. AIDS is the disease of shame. Whoever gets AIDS is considered as someone who has betrayed his or her family. Discrimination and rejection are often the next steps. Stigma and discrimination are powerful forces that have the twofold effect of demeaning individuals infected by HIV and of making it more difficult to deal effectively with the disease [8]. Stigmatization is very divisive because it separates society between “us” and “them” at a time when we actually should join hands to fight the pandemic. Stigma changes the way people view themselves. Self-stigmatization is an important challenge with which PLWH are confronted. The internalization of social rejection can lead the stigmatized individual to develop feeling of fear, anguish, shame, dejection, self-doubt, guilt, self-blame and inferiority [8]. Stigmatizing behavior and discriminatory attitudes create a culture of silence and denial, making it difficult to take the necessary action to respond effectively to HIV [8]. PLWH are not lesser human beings; they ought to be treated as dignified beings deserving the same respect as others.

After discovery of their status, many individuals experience rejection and feelings of low-esteem. Social perceptions of AIDS, in terms of collective shame and immorality, challenge the sense of communality that exists in most African societies. This happens because the community’s identity is perceived as challenged by HIV/AIDS. In Chad, as in many African societies, human identity is determined by the social web of relations in which the individual is involved. Our common humanity is defined through our relationships and not in a solipsist manner as in the western world. An individual does not exist in isolation but always as a being-in-relation. John Mbiti expresses the African communal spirit in this way: “I am because we are; and since we are, therefore I am” [10]. This corporate identity borne by every individual needs to be protected and promoted by all members of the clan. The promotion of the family/clan identity is at the core of the so-called “African solidarity.” Solidarity is both a chance and a risk. It is a chance because the commitment of the clan to the welfare of all its members creates an effective space for human flourishing. Solidarity is a risk because it demands the participation of all members in the welfare of the group. Whoever behaves poorly compromises this identity. Consequently, such a person has to be excluded to preserve the family’s reputation. Family rejection is one of the greatest evils that can be done to an individual, because it implies the loss of identity and may result in negative psychosocial effects on PLWH. Since AIDS is perceived as a shameful reality, the family may feel that it cannot compromise its identity for the sake of a misbehaving individual. Even though acceptance of PLWH has significantly improved through health education, Chad still has a long way to go.

To reduce incidences of discrimination, the parliament passed Law 19/PR/2007, the so-called “N’djamena Law” [3]. A decree that supported its application was designed in 2009 [3]. The N’djaména Law narrowly portrays HIV infection as a problem pertaining to the individual whose rights need to be protected. Anti-discrimination laws will have a better outcome if they are framed from the public health standpoint and take into account the social dimension of HIV infection. The law may prevent a member of society from ostracizing a person living with HIV, but will not address structural drivers of the infection. Population-based programs that provide public education campaigns to counter HIV/AIDS stigma and discrimination and to promote acceptance of PLWH need to be implemented both in rural and urban areas.

#### ***Human worth and ART access differentials between rural and urban areas***

In northern and eastern regions, PLWH have less access to ART compared to those living in southern regions, where there are a significant number of private and faith-based healthcare institutions. Within regions, access to ART follows the urban–rural divide prevalent in most African countries. Cities and towns are more resourceful in preventing and managing diseases than are rural areas. Treatment services are often well established in accessible areas and in the main urban areas where beneficiaries are likely to be educated and of average income or higher. In order to respond to rural AIDS, the MPH should decentralize HIV and AIDS service delivery from the district level to healthcare centers. As part of this practice of task shifting, the MPH would transfer some of the duties now pertaining to physicians to nurses. Besides ensuring that nurses are well trained to respond to rural AIDS, the District Health Commissioner should ensure that the community system is well established in all rural health centers so as to foster both community participation in public health outreach in villages and service delivery through home-based care. Home-based care has proven to be an adequate response to challenges raised by rural AIDS care. Through home-based care, community health workers can get the necessary medicines or preventive interventions to those who need them. Through the same means, patient compliance can be improved. ARVs and related services may be free, but paying transport to the district hospital, where treatment can be provided, may be something that the poor cannot afford on a regular basis [8].

#### ***Human worth and insufficient access to ARVs by children and women***

The number of PLWH has increased from 63,400 in 2006 to 206,467 in 2010 representing a 3.26 increase in 5 years. Since December 2006, efforts have been made to encourage early detection through PMTCT and voluntary counseling and testing (VCT). The important increase observed from 2006 till today has placed an extra burden on the healthcare system and on the government’s budget [3]. However, for the last 4 years, significant efforts have been deployed to provide all eligible HIV-infected adults with ARVs. The number of infected individuals receiving ARVs has significantly increased by 6 from 5,500 in 2006 to 32,832 in 2011 [5]. These significant efforts have been challenged by the increasing number of incident cases. In 2009, less than 50% of those in need of ARVs actually had access to them. While considerable efforts have been deployed to ensure that PLWH are placed on ARVs, most children and women in need of ARVs do not have access to them. The proportion of women (above 15 years old) infected with HIV represents 65% of all the infected adults. This proportion increased significantly from 2006 to 2009, from 63% to 65%, with a peak of 69% in 2007 [3]. As evidence of the disproportionate vulnerability to HIV and HIV infection in women mounts, women’s access to ARVs remains limited compared to that of men.

The number of HIV-infected children who have access to ARVs is particularly low [3]. In 2007, only 0.82% of children living with HIV were on ARVs. This situation has not improved significantly, since only 9% were on ARVs in 2009 versus 4% in 2008. Even though there has been an increase in the proportion of children on ARVs from 2006 to 2009, there are still many children living with HIV who have no access to ARVs. This situation will worsen since the estimated number of new pediatric HIV infections for children under 5 in 2010 was 656 and the prevalence of HIV infection in the same population group for 2010 was 11,965 [1]. Some of these children live in areas where even infected adults find it hard to access ARVs.

Very few medical practitioners are trained in HIV pediatric care. Access to AIDS care is determined by gender and age. When confronted with a deadly disease, society needs to ensure that all PLWH have equal capability to access ARVs. Access to ARVs should not be a privilege enjoyed by a few but a right that every infected individual should enjoy. The worth of women and children is violated when the odds of having access to ARVs are greater for men than women and for adults than for children.

#### ***Freedom from want: the AIDS and nutrition debate***

The AIDS and nutrition debate has the great merit of underlining the centrality of the human right to food, a right clearly affirmed in Article 25 of the Universal Declaration of Human Rights, which refers to the right to health [8]. AIDS treatment should be associated with good nutrition. In the course of AIDS treatment, demand for food becomes greater as the CD4 cell count increases. Such an increase requires extra food, which some people cannot always afford. Furthermore, some medications can be taken only on a full stomach, while some ARV side effects are reduced by having adequate nutrition [11]. Thus, nutritional support is vital, as a lack of adequate food security could determine whether people remain on treatment.

Food insecurity is not an alien reality in Chad. Chad is a food-insecure country. Those who live in rural areas are the most affected by food insecurity. Between late 2009 and early 2010, only 4,535 HIV-infected individuals were counted among the poorest of the poor who received nutritional support from various institutions. This number represents less than 3% of PLWH. In the context of persistent food insecurity, the increasing number of HIV incident cases undermines the country’s ability to attend to the nutritional needs of PLWH. Under international law, the state has the obligation to ensure access to food to those who cannot afford it [12]. Unfortunately, as we go through the national documents on HIV/AIDS, bearing in mind that Chad is a food-insecure country, we are shocked by the lethargy with which food security and nutrition issues have been introduced into the national discourse on the pandemic. Even though the Chadian government and its partners cannot totally fulfill the right to food, the national government should, at least, ensure that efforts are deployed to achieve progressively this basic right which is tightly connected to the right to health. Progressive realization requires that the state take steps continually to improve conditions for the realization of this right and of all rights. The concept of progressive realization also denotes the duty of the state to take immediate steps and concrete measures to realize effectively the right to food guaranteed to all.

#### ***Health***

The Constitution of the World Health Organization (WHO) defines health “as a state of complete physical, mental and social well-being” [13]. The writers of the Constitution were clearly aware of the limitations of the biomedical approach to health, through which health is defined as a state dependent on the presence or absence of disease or infirmity. Although the WHO definition has been subject to controversy, in particular as lacking operational value and because of the problem created by use of the word “complete”, it remains the most enduring [14,15]. This definition moves us beyond both the biomedical paradigm and medical individualism to underscore the way other dimensions of social and human life impact health. Health is connected to other spheres of human life and can be viewed as intertwined with other spheres of justice [2]. The context in which an individual lives is of great importance for his or her health status. It is increasingly recognized that health is maintained and improved not only through the advancement and application of health science, but also through the efforts and intelligent lifestyle choices of the individual and through the social conditions within which people carry out their daily activities [16].

The main determinants of ART access are to be found outside the healthcare sector. The major inequities are attributable to socioeconomic factors and to differences in geographical accessibility of primary healthcare facilities. These inequities are also strongly conditioned by the segmentation of health systems. ART access does not depend solely on the availability of trained AIDS care specialists and prevention specialists, the provision of ARVs, ability to treat opportunistic diseases, and AIDS-related medical technology. Being a rural dweller or living in certain region or village of the country (geographic barriers), being able to eat well or to afford the cost of care (financial barriers), being an adult or a child, being a male or a female (cultural barriers), and many other non-clinical factors determine infected individuals’ health and/or ability to access ART.

Access to primary care is important because it provides PLWH with the opportunities to avoid opportunistic diseases or to be cured from them at their early stage. Failure to have a sexually transmitted infection treated leads to persistence of important co-factors for HIV infection. Failure to treat active tuberculosis causes rapid progression of HIV. Access to care and the rate of utilization of health services are quite low due to geographic and financial barriers. Thus, access to ART is simply impossible in some areas of the country. Most of those who live with the virus have not cultivated the habit of going for check-ups on a regular basis, since they may not be able to afford basic care. As a result, these individuals may wait until their next appointment at an ART center while keeping undiagnosed conditions that may weaken their immune systems.

In order to understand the Chadian AIDS pandemic and the limited success of the universal access policy, it is necessary to highlight the non-negligible contribution of the poorly functioning public health system. The system faces many challenges that place all sorts of barriers to care. Increasing primary care coverage is crucial to improve access to health care goods and services. Access to health services is a key component of economic and social rights. To the extent that society can guarantee all its members adequate care with regard to the vicissitudes of health, it can advance towards the actual materialization of those rights and enforceable entitlement to them. Achieving this gives citizens a stronger sense of protection and of belonging to the community. Even though access to healthcare is a right, only access to ART and emergency care is given for free in Chad. Instead, access to quality healthcare depends on one’s ability to pay and on one’s geographic location. The removal of access barriers may improve people’s health status and well-being in general. Health is not just a state, but also a resource for everyday life [17].

#### ***Ability to avoid preventable death from AIDS***

Freedom from preventable death is an important dimension of well-being for PLWH, since it determines the very possibility of their exercising freedom in other spheres of human life. AIDS deaths may have declined from 14,000 in 2006 to 13,000 in 2009 in PLWH and from 11,000 to 9,600 in adults over 15 years in the general population within the same period. Thus, between 2007 and 2009, 1,000 AIDS deaths may have been avoided in PLWH and nearly 1,400 in PLWH who are older than fifteen [3]. Although the AIDS-related mortality rate has decreased as compared to 2007, when access to ARVs became free, there are still a lot of preventable deaths among PLWH. The majority of these deaths are simply not understandable, since they are completely preventable with the tools available to many people in the world [18]. These deaths are a great injustice and a stain on the conscience of society. They should be the primary object of discussion and debate within circles of health specialists and policymakers. Why is it that a disease now termed a chronic condition is still a real death sentence for many in Chad? We are faced here with an unacceptable injustice that questions the morality of modern medicine, the national distribution of means to prevent and manage diseases, and the *ethos* of local policymakers.

Freedom from preventable death can only become instrumental in promoting PLWH’s well-being if access barriers are systematically addressed. Beyond death, a genuine human sensitivity will not rest on disease management but will include prevention of both disease and unjustifiable sufferings. HIV prevention, for example, needs to be improved. Besides, there are still people who have not heard about HIV/AIDS; some do not even know how AIDS is contracted.

#### ***Access to life-saving information***

As a dimension of well-being, access to information is important because it provides individuals or population groups with the knowledge needed to refrain from high-risk behavior or to prevent unnecessary suffering. Good information passed on through persuasive communication tools, and coupled with structural interventions, can indeed be wonderful ways of encouraging behavior change. Knowledge is an important means of HIV prevention, both for MAPRs and for the general population. Access to sound information is critical in addressing social, cultural and religious misconceptions about risk perception, disease severity, and the benefits of staying on treatment. These factors also determine, directly or indirectly, clinical attendance. Believing that God or alternative medicine can cure HIV can also influence treatment patterns.

The 2009 “Cartographic and serologic study among sex workers in Chad” launched by UNAIDS showed that women from this specific group still do not have access to adequate information about modes of infection, means of prevention, and AIDS care [4]. For this population group, the message of prevention based on fidelity simply does not work. Yet, this is one of the MARPs with a very low risk perception (87.5%) sustained by an overall high risk-behavior tendency, except in Mongo (93.3%). The rate of behavior change in Sarh (60%) and N’Djamena (68.9%) remains low as compared to Mongo, Bongor, Doba, Koumra, Kelo and Moundou where the rate is above 80% [4]. Risk taking in big cities and major trade centers is quite high. It is often associated with a high level of alcohol drinking in the general population, with the low socioeconomic status of sex workers, and with a high concentration of men whose work has taken them away from their families.

The results of this study in Sarh and N’Djamena are alarming, bearing in mind the efforts and resources that have been deployed to prevent poor and risk-taking behavior. In Ati (20%), Oum-Hadjer (42%) and Mao (47%), sex workers still find it hard to change their behavior [4]. The same study shows that only 4.8% of those surveyed have a thorough knowledge of HIV. The extent of knowledge of sex workers on HIV transmission remains very poor. Most of them combine incorrect and correct information. Some still believe that HIV can be acquired through mosquito bites (34.3%), sharing food with an HIV-infected individual (32.5%), or the effect of witchcraft [4]. The lack of education cannot convincingly explain the persistence of risk-taking behaviors in this population group. The persistence of risk-taking behaviors is more an issue of both access to the right information and gender inequality wrapped with feminization of poverty. Even though the exposure level is not the same for women in the general population, social factors that structure the risk for infection do not differ tremendously from those affecting women sex workers.

In the general population, women with higher education degree are six time more likely to be infected than those who attended “medersa” (Islamic school), 1.5 times than those with only primary school education, and 1.7 times than those with no more than secondary school education [4]. These rate differentials are quite surprising. They can be explained by the fact that women with university education are more sexually emancipated than others, in a context where women’s access to available resources is constrained by gender inequality, recourse to preventive measures are underutilized, and women are seen as inferior to their male counterparts. Society, especially in northern and eastern regions, barely tolerates sex education programs. Women’s participation in HIV prevention campaigns is perceived negatively. HIV infection finds a home in women in a society that denies them access to resources and to symbolic capital. Women often lack access to cash, land and jobs [8]. Nobody would dare to question the empirically verifiable assertion that gender inequality and poverty are the strongest drivers of HIV infection in women.

#### ***Participation***

Participation is an important dimension of well-being. It is the prerequisite for social activism and advocacy through which the voices of PLWH can be heard. A democratic system emphasizes the need for social participation in decision-making processes and requires that people be empowered to participate directly or indirectly in making society a better place. Empowerment can be done through formation, financial assistance, structural intervention, and capacity reinforcement by bigger entities such as national NGOs, the state and bi- and multi-lateral donors.

The HIV/AIDS prevention project accompanying the asphalting of the N’Djamena-Doba-Koumra-Sarh road focused on community-based organizations’ ability to transform their social environment. This project aimed at changing the community’s perception about sexual relationships. Encouraging results were achieved: 126 selected peer educators and 8 supervisors were trained; 21 facilitators of community conversations and 8 supervisors were trained, and, finally, 13,492 vulnerable individuals living along the road N’Djamena-Sarh were educated [3]. To these data one may add efforts made with the subsidies of the Global Fund, Round 3, that supported interventions aimed at reaching out to 373,743 young people aged 15–24, training 3,900 peer educators, and providing 7,321 sex workers with health education [3].

The project essentially focused on community conversion. Community gatherings often took place after popular events or in popular places (funerals, burials and weekly marketplace). Conversations held in places where people gather aimed at changing people’s risk perception and at pointing out the contribution of culture and society to the pandemic. These conversations significantly reduced risk-taking behavior and increased respect for women. So far, NGOs and community-based associations have played an important role in the fight against the pandemic. However, the need for community-based advocacy has not yet created forums of discussion with other constituencies within which the concerns of PLWH and MARPs can be voiced to higher authorities. Neglecting participation as an essential dimension of well-being may have implications for policy design. Taking into consideration the unique experience of infected and affected individuals may prevent policy-makers from designing policies or legitimating programs that are not relevant to the concrete situation of those individuals.

As a political concept, participation presupposes the recognition of the substantive and instrumental roles of freedom, the need to empower disenfranchised populations, and the necessity of supporting social relations and political arrangements required to sustain and expand that empowerment. Advocating for a just society and promoting universal access to ART are not inimical. Advocacy initiatives are much needed to challenge government’s inadequate financial contribution to the healthcare sector and government’s low-level commitment to the Abuja Declaration, to address the lack of an effective and strong public health leadership in a country with poor health indicators, and to implement the “N’djamena law” on anti-discrimination of PLWH.

Free access policy has considerably reduced the economic barrier to ART in many regions of the country, but the funds needed to reinforce and to intensify programs are still lacking. The overall healthcare system is underfunded. In Chad, as in many other African countries, civil society and associations of PLWH have to keep reminding their respective governments that they have to live up to their Abuja promise. In their Declaration, African leaders pledged to commit at least 15% of the annual budget of their country to the healthcare sector [19,20]. So far, the Chadian government has committed not more than 6% of its annual budget to the health sector [7]. According to the Finance Act of 2007, the share of the budget of the MPH, compared to the general budget of the country, decreased from 8.4% in 2003 to 4.2% in 2006 and moved to 5.3% in 2007 [5]. Despite this low level of financial allocation to the health sector, the actual execution of the MPH budget varies each year: 86.9% in 2003, 85.8% in 2004, 65.4% in 2005 and 75.6% in 2006 [5]. These figures show that budgetary commitment to the healthcare sector remains quite low [5]. Civil society has the duty to challenge the underfunding of the healthcare sector and to call on the government to live up to its promise. Challenging the government to enforce the N’djamena Law (Law 19/PR/2007) in order to protect PLWH against discrimination is equally important [3].

Public health advocacy is a risky enterprise when the government does not always respect human rights and freedom of speech. Yet, civil society in Chad ought to hold the government accountable for what it promised to the entire world. Civil society can also play a key role in asking for effective public health leadership and in ensuring that the universal access policy is accompanied by programs and actions that sustain its implementation and make its effects felt among rural and vulnerable individuals. Through their collective agency, civil society organizations can hold institutions in charge of drug supply and AIDS management accountable for their actions and policies.

### A new ethical vision and effective public health responses to HIV and AIDS

Our understanding of access barriers as an issue of well-being does not corroborate the idea of AIDS as a purely biomedical problem. To promote well-being genuinely, a broad intervention is needed, since these barriers are common to access to the care required to address most diseases. It is then important to develop a new ethical vision through which health promotion can be understood as both a social issue and a development issue. There is nothing wrong with underlining personal barriers to access, but there is something deeply unfair and pernicious about using personal responsibility as the basis for assigning blame, while simultaneously denying to those who are being blamed the opportunity to exert agency in their daily lives. The macro-barriers to ART access cannot be undermined for the sake of libertarian claims of responsibility. It is only within an ethical vision that sets the stage for health promotion and social transformation that access barriers can be truly eliminated. Public health intervention is a way of doing justice, a way of asserting the value and priority of all human life over anything else [21].

The public health response to the HIV and AIDS crisis cannot be reduced to the use of analytical tools and technical solutions available to specialists [21]. An AIDS response that is not embedded in advancing social justice and promoting human rights is simply doomed to failure. Responding to HIV and AIDS is intimately connected with the practice of social justice. The limited success of the universal access policy is testimony to failure to take sufficient account of the numerous justice and human rights strands with which HIV and AIDS are intertwined [8]. Without being pessimistic, HIV and AIDS will not be uprooted if the fight against unjust structures is not given priority, because the shape and the extent of the pandemic are determined, directly or indirectly, by these structures which are often located outside the areas addressed by HIV and AIDS interventions. Actions aiming at promoting justice can be directly or indirectly equated to actions against the pandemic. Hence, macro-AIDS policies and interventions should necessarily include the fight against structures that favor the spread of the virus and maintain barriers to access to ART. These structures include all legal arrangements and social institutions that sustain and perpetrate unjustified income inequality, gender discrimination, stigma and discrimination against PLWH, and economic exclusion of citizens on the basis of ethnicity or class.

As a public health measure, ART provision calls for the adoption of a new ethical model that challenges the current social order [21]. The public health vision is essentially social and ethical. It heightens consciousness of the manifold forces threatening human life, and it requires thinking about and reacting against unnecessary suffering as primary problems of the entire society. Policies that focus on removing barriers to access may result in considerable reduction of social inequalities. The universal access policy should be provided with social rootedness and with the justice component inherent in any genuine public health intervention. Rootedness and justice are instrumental for addressing the structural roots that prevent access. Without these two important elements, universal policy will be another instrument of injustice.

#### ***Strengthening health systems***

AIDS has challenged health systems in a manner that is essentially different from other health problems. AIDS cannot be managed without a good primary healthcare system that actually brings health services to local communities which need it more. In addition, sustaining long-term treatment and prevention coverage appears to be major challenge to the healthcare sector because it entails a life-long commitment to combat the pandemic by a country that has limited resources. For some experts in public health ethics, universal health care can respond to the need for the sustained commitment to the HIV/AIDS fight. James Keenan has recently noted that “the more we respond to HIV/AIDS, the more our health care agenda inevitably expands [22].” Thus, addressing the pandemic brings about many other challenges “suggesting that now is the time to include universal health care in our HIV/AIDS conversations if we have already not yet begun to do so [22].” So far, access to emergency care is being granted for free to every citizen. However access to this life-saving measure is limited by all the factors that prevent effective and equitable access to ART by those who need it. There is an ongoing debate on the relevance and feasibility of universal health care in Chad. Even though this debate is still in its infancy, there is growing awareness in the local public health community of the need for a comprehensive solution to healthcare and to public health problems with which the entire country is confronted. Universal access to health care, which presupposes the reshaping health systems as a whole, appears to be the bedrock of such a solution.

The commitment of the Chadian government to improve healthcare infrastructure has been important in the last decade. Centers that provide care to PLWH have significantly increased, from 26% in 2007 to 32% in 2008 and to 76% in 2009 [3]. Capacity-building activities have been implemented in a way that renders AIDS care centers more operational. Until 2009, 207 prescribers were trained in the medical management of AIDS [3]. These efforts have increased geographic availability of services in AIDS care centers. Undoubtedly, despite the encouraging results achieved by public health policies, Chad faces a major challenge to improve health equity and to reduce exclusion from its healthcare system. The endemic scarcity of resources with which the country is confronted plays a role in these problems, but Chad is also wrought by shortcomings in the way the health sector is organized and governed. The limited success of the universal access policy is due, at least partly, to the weaknesses of health systems. Key elements of health systems need to be reinforced or, simply, rethought. Besides the lack of a sustained drug provision supply system, the virtual absence of a community system through which home-based care provision of both preventive and curative services can be provided to the public, poor governance of the health sector, and the insufficient number of trained clinicians and public health specialists have reduced the success rate of the universal access policy.

1) Unlike faith-based healthcare institutions, the public healthcare system does not always include local communities as stakeholders in service delivery. Local communities can play an important role in reducing loss to follow-up patients, improving access to information with regard to prevention, and supporting long-term adherence of ARVs, since AIDS is a chronic disease. ART provision in a country with under-resourced health systems poses challenges to health professionals. It forces them to think of provision in new ways, reaching beyond healthcare facilities to communities. As they attend to PLWH, health professionals have rediscovered the role of the community system in disease prevention and management, as stated in the Alma Ata Declaration and in subsequent documents on primary healthcare and health promotion [23-25]. In many church-based healthcare centers, lay community workers are trained by diocesan healthcare coordinators to perform some clinical tasks. In addition, members of the Health Committee of healthcare centers help the clinical staff with non-clinical interventions including nutritional recuperation, immunization, antenatal counseling, and public health outreach campaigns. Unfortunately, this level of organization is not found in most healthcare centers.

2) Poor leadership goes hand in hand with the brokenness of health systems. Compared to private clinics and faith-based healthcare centers, government-run health institutions are often the least prepared and the least motivated to deliver quality AIDS care. The governance of the health sector is weak, and the acceptance of the role of civil society is often seen as inappropriate both by the government and by health specialists. This situation makes real participation a challenge in most settings. Community participation is a fundamental underpinning of the primary healthcare philosophy. The concrete unfolding of the primary healthcare vision requires community participation in and mobilization around health and development-related issues. The National Health Policy recommends the creation of health and management committees in all healthcare centers throughout the country. Through the work of these two committees and that of community-based associations, health centers can be provided with an effective leadership to prevent and combat AIDS at the grassroots. Unfortunately, only a limited number of healthcare centers abide by this prescription of the MPH. The MPH needs to design regulatory frameworks, guidelines and clear policy messages to ensure and to support real and effective community participation. Involvement in decision making and in service delivery obliges PLWH and communities to be literate in both HIV prevention and AIDS treatment, as well as in how health systems work.

3) Human resources represent an important component of health systems. The absolute shortage of trained health care workers is a major impediment to treatment access and needs short-term action linked to long-term measures. Proper training for health care providers and staff is important, but not sufficient – the Chadian health care system is faced with a crisis of ever increasing patient volume that has a strong tendency to overwhelm the available human resources and infrastructure. Ensuring that trained human resources and infrastructure are appropriately proportionate to the patient load appears to be a challenge that policymakers cannot avoid.

The current state of the health systems reduces the chances of success of universal access to ART. The universal access policy cannot yield great results with a poorly performing primary healthcare system, the entry gate of the healthcare system for those seeking health services. Weakness in one component of health systems not only constrains the functioning of the system as a whole, but also limits the potential for scaling up service delivery and interventions. Health systems need to be reinforced to ensure that the national AIDS program and other major public health initiatives can achieve desired outcomes [26]. With the technical and financial support of all stakeholders and the state’s partners, the MPH needs to improve health systems. The MPH may intervene to strengthen the capacity of training institutions so that they produce better qualified health professionals and improve the management of healthcare institutions as well as of public health programs. The MPH’s intervention may focus on improving staff motivation and retention, thus reducing the brain drain of medical professionals [26].

The challenge of providing ART to those in need has brought to the forefront the lack of human resources with which Chad’s healthcare sector is confronted. The challenge of providing long-term care and prevention with limited resources is a heavy one. However, implementation of the task-shifting policy can allow the country to tackle HIV and AIDS even with limited human resources. This approach to human resource management applied to health sector is not new. Task-shifting has existed in Zambia since 2004 [27] and was implemented in South Africa in May 2010 [28]. Task-shifting consists in training, and then permitting, lesser-qualified health workers to perform tasks which they were previously unqualified to do. More concretely, it consists, for example, in allowing nurses to perform some of the tasks of doctors and lay community workers some of the roles of nurses. Such a shift could eliminate some barriers to access and could improve adherence to treatment, especially of those living in hard-to-reach areas. Task-shifting may complement the integration of AIDS care within primary care and, especially, within antenatal care to prevent MTCT. So far, in Chad, only a trained medical doctor can prescribe ARVs and some specific tests relevant to follow up of PLWH. With the implementation of task-shifting, nurses who have received adequate training can successfully provide care to PLWH. If transfer of task is accompanied by adequate training, quality of care would not be compromised and care could be more cost-effective than the present division of labor [29,30]. However, as a study in Uganda has shown, a transfer of tasks should always be accompanied by adequate training [31]. The implementation of this policy may require government commitment to improvement of both the community system and the number of trained health professionals.

In addition to efforts to improve health systems, the MPH may ensure that the interventions of the various actors abide by a code of ethics drafted by experts in health systems research and public health ethics. Ethical values of justice, transparency, competence and accountability can be the leading values of such a code. These values are ethical demands of functioning health systems. Mutual accountability can be understood as a way of fighting corruption and misuse of funds in the health sector. Ensuring that government and partners have transparent frameworks for assessing and monitoring progress in national health development strategies and in health sector programs is important for health promotion initiatives. Furthermore, having agreed commitments on aid effectiveness is important for the progress of the health system. Provision of oversight through collaboration and coordination mechanisms across sectors within and outside government, including the civil society, is essential to influencing action on key health determinants and on access to health services, while ensuring accountability.

#### ***Understanding AIDS as development issue***

The story of AIDS in Africa, as in Chad, is a story of poverty, social division and poor management of national resources. The pandemic reveals an ongoing process of malfunctioning which results in an endless disenfranchising by and within the DNA of the social, political, economic systems in Africa [32]. Africa’s social, economic and political immune systems are deeply compromised. The virus that causes AIDS is certainly a big threat, but the social environment within which infection occurs is pathogenic. Unless the determinants of ART access and the features of the environment are taken into account, the universal access policy will yield limited results. Addressing these factors calls for new policies and programs grounded in equity and informed by the complex situations in which MARPs and the general population find themselves.

Equity in access requires that we address those disparities that are deemed unnecessary, avoidable and unfair [33]. This ethical value is inherently normative, is grounded in the ethical principle of distributive justice, and is consonant with human rights [34]. It refers to the absence of unfair disparities between different social groups, such as the poor/non-poor, men/women, adults/children, urban dwellers/rural residents. Disproportionate access to ART treatment points to the way society treats socioeconomic injustices. Injustices are often treated as problems to be managed instead of being perceived as indicators of society’s moral disruption, which calls for the transformation of power relations and of mindsets which give rise to unequal access or to increased risk of infection for some and not for others.

With disproportionate access to ART, we are faced with the fundamental denial of the dignity and rights of some members of society, such as women, children and other people already facing socioeconomic exclusion. Access to ART is a basic necessity for PLWH and has a preventive effect at the population level for non-infected individuals. It cannot be understood as a matter of fortune or luck. No one in a just society should be deprived from what is needed to avoid premature death and preventable diseases. Access to ART is cost-effective, first, because preventive medicine is more cost-effective than curative services; and second, because widespread access to ART will significantly reduce incident cases and result in less spending on AIDS treatment [35]. Good ART coverage is for the common benefit. Universal access to ART can become an opportunity to heal social inequities that prevent some from enjoying their intrinsic dignity. Provisions need to be made to address the lack of pediatric ART and the limited access of women and rural citizens to ART.

Issues of distributive justice have long been debated in health policy, but the practical challenges of the distribution of ART are relatively new. Access to ART brings about a multi-faceted challenge to many sectors of society. Thus, a mere distribution of ARVS is not enough. Similarly, a policy that ensure access to ART but does not truly address access barriers is not enough either.

The problem of HIV/AIDS in Chad is not just a health issue but fundamentally a development challenge. The disease exacerbates existing problems, such as poverty, food insecurity and shortage of skilled manpower. The main consequence of this calamity in many affected countries is the reversal of the social and economic progress made during the last few decades, coupled with the serious negative impact both on households and relevant organizations and institutions. The fact that most economies in Central Africa are weak and largely dependent on donor funding further aggravates the situation. Addressing upstream determinants of health such as poverty and the lack of education requires that the state deploy efforts to build in-country capacity, to reduce poverty, to reinforce health systems, and to strengthen preparedness for disasters. These are essential elements of a sustainable health strategy. Developing strategies that concomitantly promote population health and development would be successful if the response to broader health determinants were to be accelerated.

The MPH should empower each Regional Commissioner Health to implement parts of the National Health Policy that can boost primary care both at the district and community levels. The Minimum Package of Activities (MPA), which represent the range of activities that healthcare centers can and must provide, and the Complementary Package of Activities (CPA), which are entrusted to a district hospital, are the two major strategies for effective primary healthcare delivery in Chad [6]. Unfortunately some of these activities which are amenable to integration with ART are often not implemented. Some of these activities include the development of a community system through which home-based care and public health campaigns can be organized, the implementation of “advanced strategies”, which are the local expression of public health campaigns, outreach activities from the healthcare center, and the progressive integration of ART—at least—at the district level. Even what has been prescribed has not been fully implemented.

## Conclusion

More than thirty years into the AIDS pandemic, we have learnt a lot. Partial knowledge or truncated analysis of the causes of infection and of treatment failure is simply not acceptable. There is evidence of a widespread reliance on behaviorist, cognitivist or culturalist reductionism by which approaches to HIV and AIDS response are often confined. No such reductionism can fully account for what actually happens. Prevention and care remain one-sided when they focus only on behavior change, knowledge acquisition or cultural beliefs. The universal access policy in Chad is now unfortunately constrained by these reductionist approaches. Not taking sufficiently into account the fact that the conditions limiting or promoting the AIDS response are deeply embedded in the social fabric reduces the effectiveness of this policy. We cannot help but strongly affirm that access to ART demands a broad biosocial approach. One of the lessons we have learnt from the AIDS pandemic is that success in responding to pandemics cannot ignore dimensions of well-being such as the worth of the human person, ability to avoid premature death and unnecessary suffering, freedom from want, access to life-saving information, the justice of social institutions, community participation and the respect for human rights.

In addition to these aspects of well-being, scaling up access to ART requires significant financial, technical, social and political support to be sustainable over time. Every effort must be deployed to address social inequalities and to improve the quality of the services provided. Strengthening and even transforming health systems may improve access to ART, a result that fragmented systems supported by a poorly performing service delivery system can hardly achieve. The universal access policy will not yield good results without a strong commitment to provide the HIV fight with a vision that considers health promotion a development issue. The 2011 increase in HIV prevalence is a clear sign of a failed public health leadership in the country. However, the unfailing commitment of the head of state is a sign of hope that prefigures the success of the HIV and AIDS program.

## Competing interests

The authors declare that they have no competing interests.

## Authors’ contributions

JA originated the article, did the research and wrote a first rough draft of the manuscript. BD worked to improve the clinical aspects of the paper. JA and BD all read the first version and contributed editorial and critical suggestions. After the first peer-review, JA made substantial revisions to the earlier draft and worked toward the final draft. They have all read and approved the final version of the manuscript.

## Authors’ information

Jacquineau Azétsop is lecturer in health policy, medical deontology and bioethics at Faculté des Sciences Médicales de l’Université de N’djamena in Chad. He also teaches “Health Promotion”, “Public Health Ethics” and “Social Aspects of Public health” in the Masters program in HIV/AIDS of the Catholic University of Mozambique in Beira. Jacquineau has published on public health ethics and bioethics topics in *Developing World Bioethics*, *Public Health Ethics*; *Philosophy, Ethics and Humanities in Medicine*; and *Concilium*. Jacquineau has also authored a book entitled: Structural violence, population health and health equity: preferential option for the poor and the bioethics of health equity.

Dr Blondin Diop is an internist with experience of large systemic diseases. As a physician, he worked as an internist for 15 years under Professor Herson at the salpétrière and with Professor Delfraissy at the University Teaching Hospital of Bicetre. Since 1998, he has been working in as an HIV physician. Since 2007 he is an International Medical Expert in Chad and he worked as a doctor while co-leading the national AIDS program. Moreover, it is technical advisor to the director of the National Reference Hospital. He has authored numerous scientific abstracts and articles. He has designed and drafted numerous AIDS policy documents in Chad.

## References

[B1] Power M Faden R Social justice: the moral foundations of public health and health policy 2006 New York/Oxford: Oxford University Press

[B2] Walzer M Spheres of justice: a defense of pluralism and equality 1983 New York: Basic Books

[B3] ONUSIDA/Conseil National de Lutte contre le SIDA Rapport de la situation nationale à l’intention de l’UNGASS 2010 N’djaména: UNAIDS Chad and the National AIDS Council

[B4] UNAIDS Cartographie et étude sérologique chez les travailleuses de sexe au Tchad 2009 N’djaména: Rapport provisoire, juillet

[B5] Ministère de la Santé Publique Cadre stratégique nationale de lutte contre le SIDA 2007–2011 2007 Ndjamena: Ministry of Public health

[B6] Ministère de la Santé Publique Draft de l’Annuaire des Statistiques Sanitaires 2009 N’djamena: Ministry of public health

[B7] Sen A Development as freedom 1999 New York: Alfred A. Knopf

[B8] Kelly M HIV and AIDS: a social justice perspective 2010 Nairobi: Paulines Publications Africa

[B9] United Nations Political declaration on HIV/AIDS: intensifying our efforts to eliminate HIV/AIDS 2011 New York: United Nations

[B10] Mbiti J African religions and philosophy 1969 Nairobi: East African Educational Publishers

[B11] Christian AID Don’t take on an empty stomach: why HIV treatment won’t work without food 2007 Accessed March 2013, http://www.christianaid.org.uk/…/dont_take_on_an_em23926606

[B12] United Nations Universal declaration of human rightshttp://www.un.org/en/documents/udhr/

[B13] World Health Organization, WHO Constitution 1948 Accessed august 2012, http://www.who.int/governance/eb/who_constitution_en.pdf

[B14] Callahan D The WHO definition of health Hast Cent Stud 1973 1 3 77 87 10.2307/3527467 4607284

[B15] Jadad AR O’Grady L How should health be defined? BMJ 2008 337 a2900 10.1136/bmj.a2900 19073663

[B16] World Health Organization The determinants of health 2012 http://www.who.int/social_determinants/en/Geneva. Accessed 12 May 2012

[B17] World Health Organization The Ottawa charter for health promotion 1986 Ottawa: Adopted at the First International Conference on Health Promotion http://www.who.int/healthpromotion/conferences/previous/ottawa/en/

[B18] Farmer P Pathologies of power: health, human rights, and New War on the poor 2003 Berkley: University of California Press

[B19] African Union Abuja declarationhttp://www.un.org/en/africarenewal/vol15no1/151aids5.htm

[B20] World Health Organization Abuja declaration ten after http://www.who.int/healthsystems/publications/Abuja10.pdf

[B21] Beauchamp D Beauchamp D, Steinbock B Public health as social justice New ethics for the Public’s health 1999 New York: Oxford University Press 101 109

[B22] Keenan JF Mombé PA, Orobator AI, Vella D Developing HIV/AIDS discourse in Africa and advancing the argument for universal health care AIDS 30 years down the line: faith-based reflections about the epidemic in Africa 2013 Nairobi: Paulines Publications Africa 63 82

[B23] Farmer P Leandre F Mukherjee J Community-based treatment of advanced HIV disease: introducing DOT–HAART (directly observed therapy with highly active antiretroviral therapy) Bull World Health Organ 2001 79 12 1145 1151 11799447 PMC2566712

[B24] Kasper T Coetzee D Louis F Demystifying antiretroviral therapy in resource-poor settings Essen Drugs Monit 2003 32 20 21

[B25] Schneider H Health systems and access to antiretroviral drugs for HIV in southern Africa: service delivery and human resources challenges Reprod Health Matters 2006 14 27 12 23 10.1016/S0968-8080(06)27232-X 16713875

[B26] Ministère de la Santé Publique Profile pays en ressource humaine pour la santé au Tchad http://www.hrh-observatory.afro.who.int/images/Document_Centre/Tchad_HRH_country_profile.pdf

[B27] Morris M Use of task-shifting to rapidly scale-up HIV treatment services: experiences from Lusaka, Zambia BMC Health Serv Res 2009 9 5 10.1186/1472-6963-9-5 19134202 PMC2628658

[B28] Callaghan M Ford N Schneider H A systematic review of task- shifting for HIV treatment and care in Africa Hum Resour Heal 2010 8 8 10.1186/1478-4491-8-8 PMC2873343 20356363

[B29] World Health Organization Task shifting to tackle health worker shortages 2007 accessed March 2013, http://www.who.int/healthsystems/task_shifting_booklet

[B30] Jaffar S Rates of virological failure in patients treated in a home-based versus a facility-based HIV-care model in Jinja, southeast Uganda: a cluster-randomised equivalence trial Lancet 2009 374 9707 2080 2089 10.1016/S0140-6736(09)61674-3 19939445 PMC2806484

[B31] Lutalo IM Training needs assessment for clinicians at antiretroviral therapy clinics: from national survey evidence in Uganda Hum Resour for Health 2009 7 76 10.1186/1478-4491-7-76 PMC2752450 19698146

[B32] Katongole E Bujo B, Czerny M An age of miraculous medicine AIDS in Africa: theological reflections 2007 Nairobi: Africa Paulines Publications Africa 104 119

[B33] Whitehead M The concepts and principles of equity and health Int J Health Serv 1992 22/3 429 445 1644507 10.2190/986L-LHQ6-2VTE-YRRN

[B34] Braveman P Gruskin S Defining equity in health J Epidemiol Comm Health 2003 57 254 258 10.1136/jech.57.4.254 PMC1732430 12646539

[B35] Smith P Rosamond R, Battin MP, Silvers A Justice, health, and the price of poverty Medicine and social justice: essays on the distribution of health care 2002 New York, Oxford: Oxford University Press 301 312

